# First detection of emerging HoBi-like Pestivirus (BVD-3) among some persistently infected dairy cattle herds in Egypt

**DOI:** 10.1007/s11250-022-03332-2

**Published:** 2022-10-07

**Authors:** Ahmed F. Afify, Rabab T. Hassanien, Hala K. Abdelmegeed, Ebtsam A. Abouelyazeed, M. H. Ali, Dina A. Abdelwahed, Tahani S. Behour

**Affiliations:** 1 Virology Research Department, Animal Health Research Institute, Agriculture Research Center (ARC), 12618, Dokki, Giza, Egypt; 2 Biotechnology Research Unit, Animal Reproduction Research Institute, ARC, Al Haram, Egypt

**Keywords:** BVD, HoBi-like Pestivirus, Persistent infection, ELISA, RT-PCR

## Abstract

Bovine viral diarrhoea virus (BVDV) is a serious veterinary health concern worldwide. We conducted this study to determine the prevalence of persistent infections (PI) and identify the current strain among some dairy cattle herds in Egypt. A total of 240 serum samples were collected from six Egyptian provinces. Between 2019 and 2020, samples were tested by Enzyme linked immunosorbent assay (ELISA) for detection of PI animals, and then molecular characterization was performed. Six calves were found PI with a prevalence of 2.5% (6/240). Using molecular characterization, HoBi-like Pestivirus (BVD-3) was successfully identified in Egypt for the first time. Based on the BVD-3 reference strains on Genbank, the detected strains had an identity ranging from 98.8 to 99.6%. Partial nucleotide sequence of the 5′UTR gene for six tested samples was submitted to Genbank with accessions: OM324396, OM324397, OM324398, OM324399, OM3243100, and OM3243101.

## Introduction

It is well known that (BVDV) causes endemic disease in cattle, which has a profound impact on the worldwide economy because of immunosuppression, ill-thrift, premature culling, and a potentially fatal hemorrhagic syndrome called mucosal disease (Khodakaram-Tafti and Farjanikish 2017). BVDV is present in most cattle-producing countries, and according to Richter et al. (2019), since 1960, 107 countries have reported implementing mitigation activities.

Even though BVDV took its name from the bovine first host, the virus still infects approximately forty species, and most wild ruminants are susceptible to BVD virus infection, according to serological evidence. Besides wildlife, various non-bovid species are also thought to carry and spread the disease, although there is evidence of transient infection (TI) within most animals, resulting in the familiar BVDV syndromes (Nelson et al., 2015; Vilcek and Nettleton, 2006). However, the risk of transmission from wildlife to cattle still needs to be determined (Uzal et al., 2016).

BVDV infection can cause subclinical infections as well as a wide range of particular clinical signs, including diarrhoea, respiratory restlessness, and decreased reproductive performance, including infertility, longer calving intervals, early embryonic death, premature birth, abortive or stillbirth, congenital malformations, as well as persistently infected offspring (PI) (Garoussi et al., 2019 and Timurkan and Aydin 2019).

The virus belongs to the genus Pestivirus, which is within the family Flaviviridae, along with classical swine fever virus and Border disease virus agents, and it has a ssRNA genome of positive polarity with a length of approximately 12.3 kb. A conserved region at the beginning and end of the genome is the untranslated region (UTR). Genes for structural and nonstructural proteins are encoded within a single ORF within the genome (Meyers and Thiel 1996).

International Committee of Taxonomy of Viruses (ICTV) has renamed pestiviruses and classified them into 11 species (Liu et al., 2019). Thus, they are classified as follows: BVDV-1 (Pestivirus A), BVDV-2 (Pestivirus B), border disease (Pestivirus D), classical swine fever (Pestivirus C), and HoBi-like (Pestivirus H). In terms of sub-types, BVDV-1 belongs to the range 1a to 1q, whereas BVDV-2 belongs to the range 2a to 2d., The recent discovery of BVDV-3 (Pestivirus H) of the Pestivirus genus, HoBi-like Pestivirus, has shown that it is genetically and antigenically found in cattle and buffalo (Mishra et al. 2014).

Foetal bovine serum from Brazil was first identified as bearing a HoBi-like Pestivirus in Germany in 2003 (Schirrmeier et al., 2004)**.** The term “HoBi-like Pestivirus” is taken from the first isolate, D32/00 “HoBi.” Several HoBi-like Pestiviruses originated from countries such as South America, Europe, and Asia and were detected in commercial cell culture or FBS batches (Mao et al., 2012; Stahl et al., 2010; Stalder et al., 2005; Xia et al., 2011). The first natural case was reported in 2006 from aborted foetuses in Brazil (Cortez et al., 2006). Since then, several naturally occurring cases of HoBi-like Pestiviruses have been reported in Thailand, Bangladesh, Italy, and Brazil (Decaro et al., 2016; Haider et al., 2014; Liu et al., 2009; Weber et al., 2016; Barreto et al., 2022).

Pestiviruses have been classified according to phylogenetic analysis based primarily on 5′ untranslated regions (5′ UTR) of their genes but also based on genes like N^pro^, E2, and NS3 (Nagai et al., 2008,and Silveira et al., 2017). According to their cytopathic effect on cell cultures, BVDV species have two biotypes, non-cytopathic (NCP) and cytopathic (CP) (La polla et al., 2022). When NCP viruses are transmitted between the 40th and the 120th day of gestation (vertical transmission), immune-tolerant and PI calves are born (Nelson et al., 2015). The foetal immune system is still immature during this period and unable to distinguish between viral proteins and self-proteins. Some PI calves may live until maturity. If they are retained for breeding, their offspring are always PI but often die during their first year due to secondary infections (Martin et al., 2016).

Since PI animals shed the virus at high titers for life in all their secretions and excretions, they are the primary source of BVDV horizontal transmission in cattle herds **(**Timurkan and Aydin 2019).

The economic implications of this disease have led several endemic countries, including Egypt, to initiate BVDV control or eradication programs. Many control programs in many countries are based on two main targets: detecting PI animals, removing them from herds, and preventing new PI animals from being introduced with biosecurity programs and/or vaccinations.

BVD viruses are globally important cattle pathogens, with a considerable economic impact due to acute infection and induced immunosuppression, also due to effort needed for control (vaccination, testing, culling). Eradication of BVDV in cattle is widely performed in all cattle-producing countries, including Egypt, but is mainly hindered by the continuous production of PI calves, which consists of a natural reservoir for the virus (Russell et al., 2020).

In this paper, we aim firstly to investigate the prevalence of PI among some dairy cattle herds to identify the main reservoir of the virus as a preliminary step for further institutional molecular-based epidemiological studies of BVDV in Egypt. Therefore, these molecular techniques not only determine the epidemiological status of the infection but also can track the source of infection and contribute to the control of BVDV infection from international trade (Vilček et al., 2003).

Identifying the circulating BVDV genotypes is critical for creating appropriate diagnostic tests and control strategies or vaccinations (Alpay et al., 2019). Therefore, we also aim to identify the current circulating BVDV strain among the cattle population in Egypt.

## Materials and methods

### Collection and preparation of samples

We collected 240 serum samples from non-vaccinated Holstein calves of different ages (1–6 months) from private dairy cattle farms in six (6) Egyptian provinces (Menya, Beni-suef, Damietta, Fayioum, Kafr-Al sheikh, and Behira) during 2019 and 2020.

The epidemiological data and clinical signs were collected and observed during recurrent farm visits. No specific clinical signs were observed; the case history of the affected farms was retarded growth of many calves, early embryonic death, and subfertility of dams.

To separate serum, a sample set was placed in clean, dry centrifuge tubes, left to clot, and centrifuged at 1500 × g for 20 min. The serum was stored at − 20 °C until further testing.

All tested animals were retested with second samples 5 weeks after the first.

### Reference BVD strains

BVDV genotype 1 Egyptian cytopathic strain (Iman strain) and BVDV genotype 2 Egyptian cytopathic strain (Strain 125) were obtained from the Department of Rinderpest like Diseases, Veterinary Serum and Vaccine Research Institute, Cairo, and used as a control strain for RT-PCR testing.

### Serological tests

Double Antibody sandwich Elisa assay (Enzyme linked immunosorbent assay) (INGEZIM BVD DAS®) was used for BVDV p80/125 antigen detection in the collected sera samples according to the manufacturing instruction.

BVD p80-125 (NSP2-3) antibodies were detected in sera samples using Competitive Elisa (ID Screen BVD p80 Antibody competition Elisa IDvet Innovative Diagnostic®), as per manufacturer instructions.

For more confirmation about the PI and to avoid errors about TI, these animals were retested 5 weeks apart (Garoussi et al., 2019).

## Molecular detection of BVDV

### Extraction and RT-PCR

The manufacturer’s instructions were followed when RNA was extracted from 250 μl of each sample and NADL reference strain as a positive control using QIAzol Lysis Reagent **(QIAGEN)**. In brief, each sample was mixed with 750 μl QIAzol reagent and extracted with 150 μl of chloroform. Precipitated viral RNA was prepared using 500 ml isopropanol in the aqueous phase. The next step was to wash the pellet with 750 ml of 75% ethanol and suspend it in 20 ml of sterile DEPC water. As soon as the RNA was extracted, it was reverse transcribed to cDNA using the Revert Aid First Strand cDNA Synthesis kit (Thermo Scientific, Germany) following the manufacturer’s instructions and stored at − 70 °C until it was needed.

A 208 bp fragment of the 5′ untranslated region (5′ UTR) of the Pestivirus genome was amplified by PCR amplification by BVD 190-F and V326 primer pairs **(**Moorthy et al., 2019**)** for the detection of BVDV in a 25 *L reaction volume. A total of 12.5 μl (2X) **Thermo Scientific™ DreamTaq™** Green PCR Master Mix (**Thermo Scientific, Germany**) was used in each reaction; 0.25 μl (25 pmol) BVD 190-F forward primer (5′-GRAGTCGTCARTGGTTCGAC-3′), 0.25 μl (25 pmol) V326 reverse primer (5′-TCAACTCCATGTGCCATGTAC-3′), 7 μl water, and 5 μl cDNA sample. The PCR tubes were placed in **the Thermo Scientific™** PCR detection system, which programmed for the test as follows: 4 min at 95 °C, then 35 cycles of denaturation step at 94 °C for 45 s, annealing at 58 °C for 45 s and extension at 72 °C for 45 s followed by one cycle of final extension at 72 °C for 10 min. Analysis of PCR products was carried out in 2.0% agarose gel electrophoresis and visualized on a UV trans-illuminator.

### Sequencing and phylogenetic analysis

QIAquick Gel Extraction Kit **(Qiagen)** was used to purify positive amplicons (in terms of the 5′ UTR gene) from the gel as instructed by the manufacturer. ABI PRISM Big Dye Terminators v3.1Cycle Sequencing Kit (**Applied Biosystems, Foster City, CA, USA**) was used to directly sequence the purified PCR products. Applied Bio-Systems, CA-USA, provided a Centrisep purification kit to clean up the sequencing reaction products. ABI PRISM3500genetic analyzer (**Applied Biosystems**) was used to sequence the purified products directly.

To identify the sequence identity, BLAST® analysis (Basic Local Alignment Search Tool) was performed (Asplund et al., 2019). In BioEdit Program v.7.0.5 (Hall 1999), raw sequence reaction data were aligned with reference strains obtained from Genbank. In order to test the significance of the phylogenetic tree, we constructed it with the maximum likelihood (ML) method and performed a bootstrap test (1000 replication) using Mega version 6 (Tamura et al., 2013).

## Results

### Detection of PI state in collected samples

All samples were serologically tested to detect BVD, both antigen and antibodies, to investigate the PI state among tested animals. Samples negative for antibodies and positive for antigen (tested twice 5 weeks apart) indicated PI animal. Six (2.5%) of two hundred and forty studied samples (6/240) were found to be PI with BVDV (Table 1).

**Table 1:** The serum samples in the study indicate the total number per governorate, results of BVDV antibodies, and results of BVDV antigen tested twice, indicating the PI animals

Governorates	Total	+ ve Abs	+ ve Ag	PI
Behira	68	19	9	2
Beni seuf	42	10	5	1
Damietta	39	13	2	2
Fayoum	30	15	3	-
Kafr Al-Sheikh	35	9	2	1
Menya	26	11	4	-
Total	240	77 32.08%	25 10.4%	6 2.5%

### ***Molecular detection of BVDV***

Six samples of BVDV PI animals were tested for molecular confirmation using oligonucleotide primers specific to the BVDV 5′ UTR described previously. The gene of the 5′ UTR was amplified, and all samples were found positive to have a specific M.W band at 208 bp.

### Sequence and sequence analysis

Partial nucleotide sequence of the 5′ UTR gene of six tested samples named: (BVD-3_AHRI_EGY_1, BVD-3_AHRI_EGY_2, BVD-3_AHRI_EGY_3, BVD-3_AHRI_EGY_4, BVD-3_AHRI_EGY_5, and BVD-3_AHRI_EGY_6) has been submitted to the Genbank with accession numbers **(OM324396, OM324397, OM324398, OM324399, OM3243100, and OM3243101)** respectively.

**Fig. 1:**
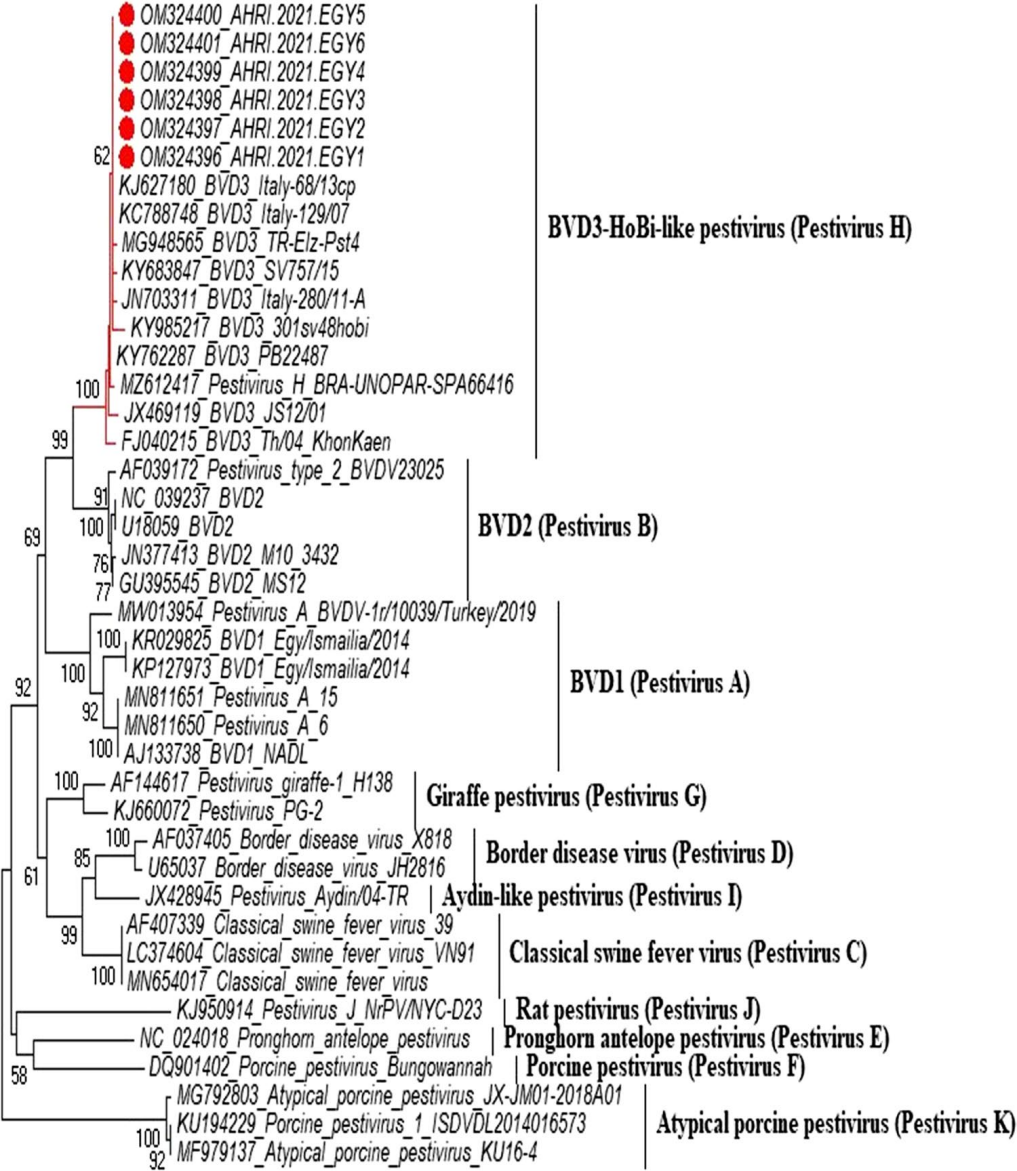
The phylogenetic tree was created by MEGA 6 software using the maximum likelihood method. The confidence level of the neighbour-joining tree was evaluated by bootstrapping using 1000 replicates. The red dots refer to the samples in our study, with their names and accession numbers

Based on the phylogenetic analysis, all six samples were atypical HoBi-like Pestiviruses (BVD-3). According to the analysis of sequence data, our strains are 99.6% similar to the Brazilian BVD-3 reference strain (**ACC. KY683847**), 98.8% identity with the BVD-3 reference strain from Italy (**ACC. MH410816**), and 99.6% identity with BVD-3 other reference strain from Italy (**ACC. HQ231763**) (Fig. 1 and Table 2).

**Table 2:**
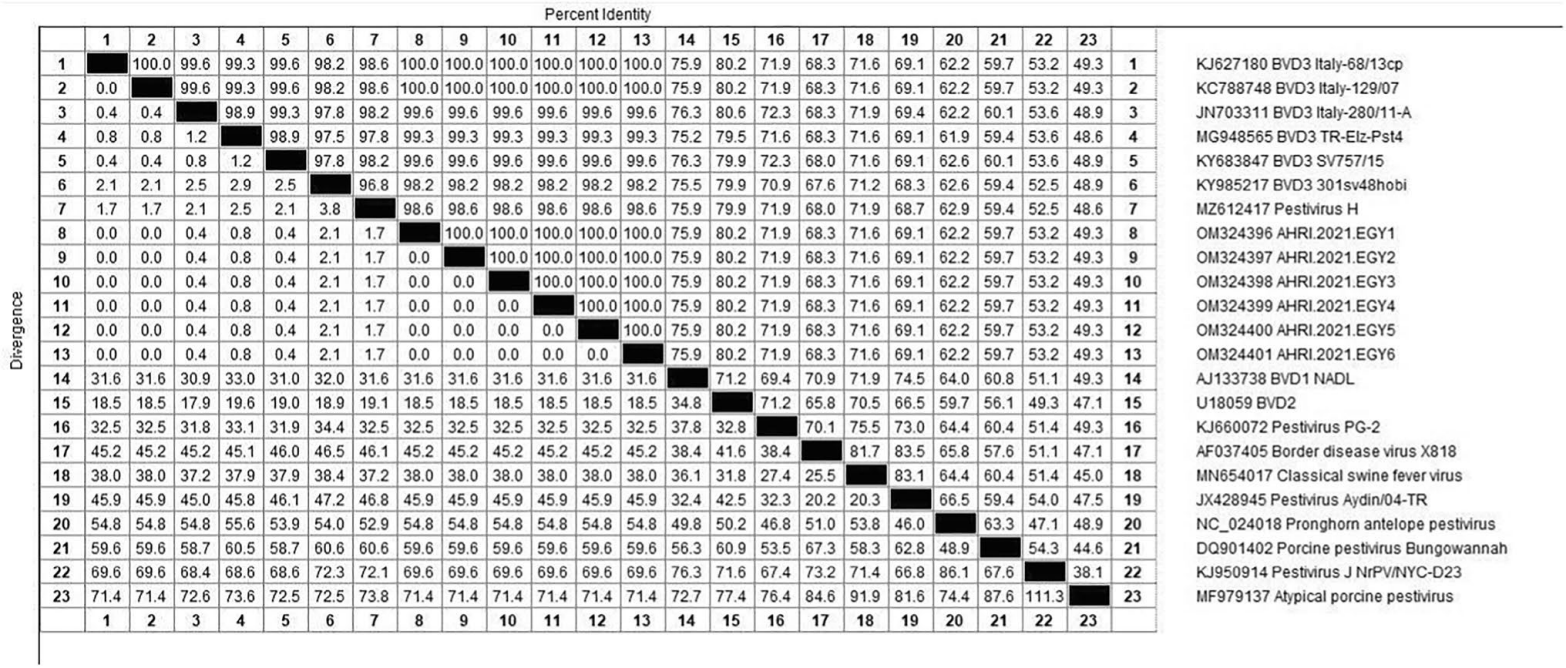
Percentage of nucleotide sequence identity between strains of the current study (BVD-3_AHRI_EGY strains) and reference strains published on Genbank based on partial nucleotide sequences of 5′ UTR gene

## Discussion

During this study, we aimed to investigate the prevalence of PI state among some Egyptian dairy cattle herds and to detect and identify the current strain circulating among the national cattle population.

The PI calves were detected by serological analysis of 240 serum samples. These samples were tested to identify seronegative (immunotolerant) animals which are positive at the same time for the BVDV antigen for two successive tests. This came in agreement with previous records, which revealed that persistently infected animals do not produce BVDV-specific antibodies (Dezen et al., 2013; Alpay et al., 2019).

In the present study, the size of the unvaccinated herd is medium-sized, and examining each animal is not strategically or financially solution for PI animals’ detection.

When seronegative dairy animals are naturally infected, no extreme illness is caused by BVDV; they have become transiently infected (TI) animals with antigens that may be detected for weeks (Niskanen and Lindberg 2003). TI animals may be a source of horizontal infection, as it is previously reported that BVDV can survive in a herd even when PI animals are absent (Moennig et al., 2005). PI and TI animals likely existed in this study, and due to the greater probability of BVD virus shedding in PI than the TI cows, the PI cows were considered the leading cause of infection in this study. For more confirmation about the PI and to avoid errors about TI, these animals were retested 5 weeks apart (Garoussi et al., 2019).

The PI calves with BVDV in the present study were detected serologically and confirmed with RT-PCR in six out of two hundred and forty studied samples (6/240), the same results reported previously by Hilbe and co-authors. They detected three PI animal cases in their research using ELISA and RT-PCR techniques for testing serum samples collected from calves infected by BVDV (Hilbe et al., 2007).

The worldwide pooled PI prevalence at the animal level ranged from low (≤ 0.8% Europe, North America, and Australia), medium (> 0.8 to 1.6% East Asia) to high (> 1.6% West Asia) (Scharnböck et al., 2018).

According to the current study’s findings, the prevalence of PI animals was 2.5%. A high percentage of seropositive animals (32.08%) indicated the presence of PI animals within the herd (Garoussi et al., 2019). Some studies reported that the BVDV PI prevalence in cattle was lower and extended from 0.5 to 2% (Brock, 2003; Peterhans et al., 2003; Smith et al., 2008). Thus, it is clear that the prevalence of PI within herds is variable, and it can reach as high as 30–35% when many naïve cows are exposed to NCP BVDV early in pregnancy (Khodakaram-Tafti et al., 2017). On the other hand, the prevalence of calves being born PI thus diminished substantially from around 1.4 to < 0.02%.in Switzerland (Schweizer et al., 2021).

Moreover, comparing the situation in Egypt, collectively, we found that the current study PI prevalence is lower compared to the latest study, where the PI was confirmed in 9 calves out of 305 calves (2.95%) in Damietta governorate in Egypt (Atwa et al., 2019). An earlier study also detected that BVDV prevalence is 8.4% of serum samples (21/250) by antigen capture ELISA (El-Bagoury et al., 2014).

Such discrepancy in the reported prevalence percentages may be due to many factors: for instance, the used diagnostic tests in each study, the variation in the management system for the examined herd, and the locations of the examined population (Atwa et al., 2019).

Moving to another factor, the herding breed might play a significant role in determining BVDV prevalence. It should be noted that most of the cattle examined in this study were pure Holsteins. As previously reported, the prevalence of BVDV in pure Holstein Friesian cows was highest (46.6%) compared to Brown Swiss (21.8%) and Creole breeds (22.7%) (Herrera-Yunga et al., 2018). Hence, the genetic factor affected cow susceptibility to BVDV infection (Ortega et al., 2020).

Considering the age factor, the PI prevalence in this study was observed in calves ranging from 1 to 6 months, which came following Smirnova et al. (2008), who revealed that at birth, the prevalence of BVDV PI is highest, and it declines with age.

As a routine diagnostic method for BVDV, RT-PCR has gained widespread use in the past decade (Smith et al., 2008). It has been shown that PCR allows for the detection of BVDV in a variety of clinical samples such as serum, buffy coats, tissues, foetal fluids, milk, and nasal swabs **(**Renshaw et al., 2000;Stokstad et al., 2003;Kennedy et al., 2006;Youngl et al., 2006;Edmondson et al., 2007;Tajima et al., 2008;Khodakaram-Tafti et al., 2016). This technique is reliable for diagnosing BVDV in all ages of PI, even with maternal antibodies to BVDV that affect the accuracy of other tests such as VI and ELISA (Luzzago et al., 2001; Saliki and Dubovi, 2004; Goyal, 2005**; **Sandvik, 2005).

Regarding genome organization of the virus, the BVDV E2 structural protein induces the production of neutralizing antibodies (Thomas et al., 2009), and the N^pro^ nonstructural protein contributes to virulence (Darweesh et al., 2018). Pestivirus strains are frequently classified using the 5′UTR. Interestingly, the new strain BVD-3 AHRI EGY detected in this study shared more homology in the 5′UTR with other strains from Brazil and Italy. This new emerging strain may be introduced to Egypt from those areas through cattle importations and also may be due to the trading of contaminated biological products (Bauermann et al., 2013).

Even though there was not enough information about the presence of HoBi-like Pestivirus in the Middle East and African countries, there were some previous reports about the widespread presence of BVDV-1 and sporadic existence of BVDV-2 (El-bahgy et al., 2018;Atwa et al., 2019;Lotfy et al., 2020;Guidoum et al., 2020;Pawlos et al., 2020; Aboezz et al., 2021 and Endeshaw et al., 2021).

HoBi-like Pestivirus infections have recently posed a concern for cattle and small ruminants in South America, Europe, and Asia. To our knowledge, there have been no clinical cases of HoBi-like Pestivirus infection in cattle in the Middle East, although the discovery of HoBi-like Pestivirus strains in contaminated cell cultures, commercial FBS, and small ruminants.

In conclusion, the current study successfully identified a new emerging atypical HoBi-like Pestivirus (BVD-3) among PI cattle herds for the first time in Egypt.

This study has some limitations. The BVDV PIs have been identified in bovine calves reared in only six Egyptian provinces and did not elucidate PI in other provinces. Consequently, it is necessary to carry out further studies to investigate the PI of BVDV in other provinces, particularly the southern part of Egypt and the delta. Additional studies are required to identify BVDV infection in other animal species susceptible to BVDV, such as buffalo calves and goats. This will help to establish a strategy for controlling BVD infection.

It was revealed that there is a correlation found between the prevalence of seropositive animals and the presence of BVD PI within the herds. Overall, to minimize the diagnostic detection costs and economic losses caused by this disease, it is recommended to pool whole blood samples for RT-PCR.

To evaluate the outcome of animal genetics on both individual and herd seroprevalences levels, more future studies with enough details focusing on the type of production system utilized by the sampled animals and their breed composition are required.

Further molecular-based analysis on 5′ UTR complete gene sequence or BVDV entire genome sequence by NGS (next generation sequencing) would investigate the evolution of quasispecies of the identified BVD strain as a preliminary step for control of PI in Egypt.

## Data Availability

All data generated or analysed during this study are included in this published article [and its supplementary information files].
